# Extracellular Vesicles-Loaded Fibrin Gel Supports Rapid Neovascularization for Dental Pulp Regeneration

**DOI:** 10.3390/ijms21124226

**Published:** 2020-06-13

**Authors:** Siyuan Zhang, Anja Lena Thiebes, Franziska Kreimendahl, Stephan Ruetten, Eva Miriam Buhl, Michael Wolf, Stefan Jockenhoevel, Christian Apel

**Affiliations:** 1 Department of Biohybrid and Medical Textiles (BioTex), AME - Institute of Applied Medical Engineering, Helmholtz Institute of RWTH Aachen University & Hospital, 52074 Aachen, Germany; syzhang1987@outlook.com (S.Z.); thiebes@ame.rwth-aachen.de (A.L.T.); franziska.kreimendahl@gmail.com (F.K.); jockenhoevel@ame.rwth-aachen.de (S.J.); 2 Electron Microscopy Facility, Institute of Pathology, RWTH Aachen University Hospital, 52074 Aachen, Germany; sruetten@ukaachen.de (S.R.); ebuhl@ukaachen.de (E.M.B.); 3 Department of Orthodontics, RWTH Aachen University Hospital, 52074 Aachen, Germany; michwolf@ukaachen.de

**Keywords:** extracellular vesicles (EVs), dental pulp regeneration, hydrogel, angiogenesis, rapid vascularization, VEGF

## Abstract

Rapid vascularization is required for the regeneration of dental pulp due to the spatially restricted tooth environment. Extracellular vesicles (EVs) released from mesenchymal stromal cells show potent proangiogenic effects. Since EVs suffer from rapid clearance and low accumulation in target tissues, an injectable delivery system capable of maintaining a therapeutic dose of EVs over a longer period would be desirable. We fabricated an EV-fibrin gel composite as an in situ forming delivery system. EVs were isolated from dental pulp stem cells (DPSCs). Their effects on cell proliferation and migration were monitored in monolayers and hydrogels. Thereafter, endothelial cells and DPSCs were co-cultured in EV-fibrin gels and angiogenesis as well as collagen deposition were analyzed by two-photon laser microscopy. Our results showed that EVs enhanced cell growth and migration in 2D and 3D cultures. EV-fibrin gels facilitated vascular-like structure formation in less than seven days by increasing the release of VEGF. The EV-fibrin gel promoted the deposition of collagen I, III, and IV, and readily induced apoptosis during the initial stage of angiogenesis. In conclusion, we confirmed that EVs from DPSCs can promote angiogenesis in an injectable hydrogel in vitro, offering a novel and minimally invasive strategy for regenerative endodontic therapy.

## 1. Introduction

Dental pulp tissue can be damaged in the case of caries by the penetration of bacteria or by a dental trauma. In these situations, the pulp tissue gradually becomes inflamed and if this inflammation is not stopped early, pulp necrosis occurs. Even after successful conventional therapy with synthetic materials, these teeth are more prone to fractures. Tissue engineering strategies have great potential for the regeneration of the pulp–dentin complex. In principle, two different approaches can be distinguished. Either an attempt is made to implant cells, mainly mesenchymal stromal cells, combined with scaffolds or a cell-free approach is pursued. In this case, bioactive materials are used to favor the colonization of the body’s own cells [1]. Both regenerative strategies are dependent on vascularization, which is needed to deliver nutrients and oxygen to cells, and to facilitate the removal of metabolic waste, CO_2_ and cell debris from the tissues [2]. Inside the tooth, the surrounding mineralized dentin wall, the narrow root canals and the tiny apical access generally restrict the blood supply. Cells implanted in the course of regenerative approaches could also not survive solely through oxygen diffusion from the surrounding host tissue. Therefore, most cells in engineered pulp tissue undergo apoptosis due to these space restrictions, and rapid induction of neovascularization is essential to ensure deposition and mineralization of the extracellular matrix (ECM) after implantation of engineered pulp [3].

Recently, extracellular vesicles (EVs) have been shown to play a key role in neovascularization. EVs are particles naturally released from the cell that are delimited by a lipid bilayer and cannot replicate. They carry a cargo of bioactive molecules (messenger RNA (mRNA) and microRNAs (miRNAs)), enzymes, cytokines, chemokines, immunomodulatory and growth factors. According to the recommendation of the International Society for Extracellular Vesicles, the term extracellular vesicles (EVs) covers a broad range of membrane nanovesicles, including small endosome-derived exosomes (diameter 30–150 nm) and large plasma membrane-derived microvesicles (diameter up to 1 µm) secreted by cells for cell communication [4]. EVs released from mesenchymal stem cells (MSCs) have been shown to promote angiogenesis and wound healing, with similar effects to their parent cells under pathophysiological conditions [5,6]. The ability of MSC-derived EVs to carry therapeutically relevant molecules that stimulate angiogenesis and tissue repair was demonstrated by the dose-dependent enhancement of endothelial cell proliferation, migration, and tube formation. This was also shown for dental pulp cell-derived exosomes that stimulated human umbilical vein endothelial cell (HUVECs) angiogenesis [7]. This finding highlighted that exosomes of dental origin might be promising biomimetic tools to apply in pulp regeneration. However, administering a therapeutic dose of EVs to target cells is not easy and has its challenges. The ability to prolong the half-life of EVs at the target site is crucial to maintain the therapeutic dose of EVs. Studies have shown that direct intravenous, intraperitoneal or subcutaneous injection of EVs results in rapid removal from the bloodstream and accumulation in the liver, spleen, lungs and gastrointestinal tract [8,9]. Regardless of the route of administration, the majority of EVs are rapidly taken up by macrophages in the reticuloendothelial system and removed from the body [10]. The retention time of topically applied unprotected EVs, e.g., on the skin or mucous membrane, could be even shorter, since the vesicles would probably be quickly removed by fluid (sweat or saliva) and external factors.

In order to apply and to retain EVs in damaged tissues, different scaffolds have been investigated. Examples include ceramic scaffolds, hydrogels, and synthetic polymers, which have been shown to promote bone regeneration, cartilage repair, and wound healing, respectively [6,11,12]. EVs embedded in injectable hydrogels could offer a simple way to preserve the activity of EVs during neovascularization and are preferred for the regeneration of dental pulp, because sol-gel conversion is convenient for implantation into narrow locations [13]. Hydrogels are characterized by a high water content (≥90%) and offer sufficient porosity for nutrient diffusion, similar to the properties of natural ECM. They also allow the incorporation and sustained release of bioactive molecules to support the formation of new tissues. Using these injectable scaffolds, pulp revascularization therapy can be achieved in situ with a minimally invasive technique that preserves tooth structures to encourage functional reconstruction [14].

**Figure 1:**
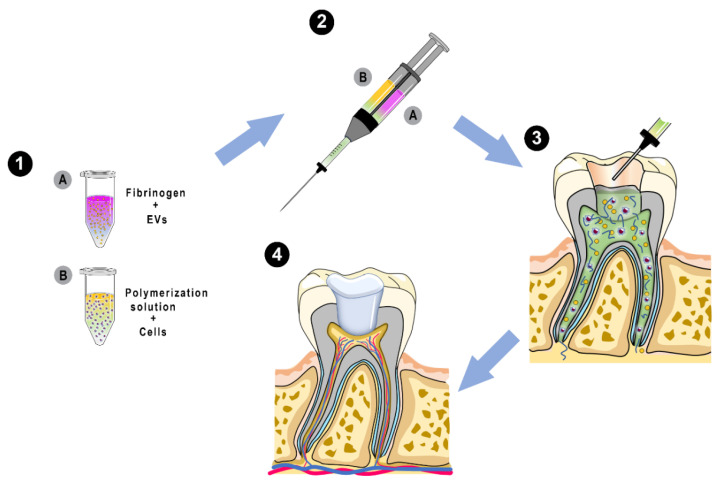
Schematic representation of the proposed in situ pulp tissue regeneration. (**1**) Preparation of the polymeric solutions for fibrin gel fabrication. Solution (**A**) includes fibrinogen and extracellular vesicles (EVs). Solution (**B**) includes polymerization solution with dental pulp stem cells (DPSCs) and human umbilical vein endothelial cell (HUVECs). (**2**) Co-injection of the polymeric solutions using a dual-barrel syringe. (**3**) Fibrin gel fits the root canal system and undergoes gelation. (**4**) Rapid formation of vascular networks in engineered pulp within several days.

In this study, we propose the development of an injectable hydrogel with enhanced proangiogenic properties as a basis for a future regenerative endodontic therapy (Figure 1). Given the well-recognized proangiogenic properties of fibrin gel [15], we decided to use this hydrogel to incorporate EVs. Using a double cylinder syringe, EV-loaded fibrin gel can be applied by co-injection of polymer solutions into the root canal system and can serve as an optimized cell carrier or as a bioactive in situ forming system. As a proof-of-concept, we isolated and characterized EVs from dental pulp stem cells (DPSCs), optimized the EV load required for fibrin gels, and evaluated the benefits of the EVs by investigating cell growth and cell migration. Co-cultures of endothelial cells and DPSCs were used to study the proangiogenic activity of the EV-loaded fibrin gel. Two-photon imaging was used to quantify vascular-like structure development, followed by the evaluation of collagen deposition and apoptosis. Our study highlights a rapid neovascularization in EV-loaded fibrin gel even under starvation culture conditions in vitro, mimicking the harsh environment of the dental pulp chamber. The described approach offers a promising solution for the regeneration of vascularized pulp tissue.

## 2. Results

### 2.1. Confirmation that Isolated Dental Pulp Cells Are MSCs

The expression of cell surface markers was investigated by flow cytometry. The mesenchymal markers (CD73, CD105) and the cell adhesion molecules (CD90) were confirmed as present in the DPSCs, whereas the hematopoietic lineage markers (CD45, CD34, CD11b, CD79a and HLA-DR) were absent (Supplementary Figure S1). These results confirmed the immunotype as consistent with MSCs [16].

### 2.2. DPSC-Derived Vesicles Display the Key Characteristics of EVs

**Figure 2:**
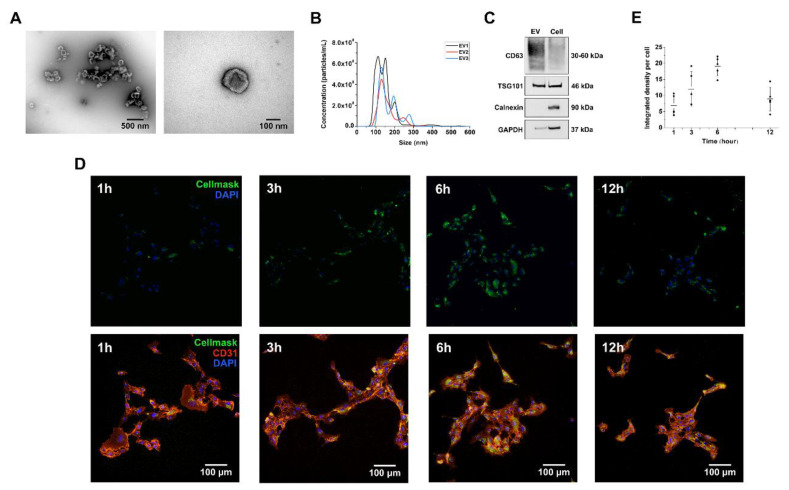
Characterization of DSPC-derived EVs. (**A**) Representative TEM images of EVs. (**B**) Size distribution of EVs was determined by nanoparticle tracking analysis (NTA), presented by three biological replicates (EV1–EV3) isolated from different DPSC cell lines. (**C**) The surface markers of EVs were analyzed by western blotting. (**D**) The uptake of CellMask Green-labeled EVs by HUVECs (stained with CD31) was visualized after 1, 3, 6 and 12 h of incubation, showing a time-dependent course. (**E**) The uptake of EVs was quantified as integrated fluorescent density signal per cell. The number of internalized EVs reached the maximum at 6 h.

Transmission electron microscope (TEM) revealed the presence of homogenous, cup-shaped vesicles that were prone to aggregation (Figure 2A). The size distribution of the vesicles was 80–200 nm as determined by nanoparticle tracking analysis (NTA) (Figure 2B), and the yield was 0.8–1.0 × 10^9^ particles per million DPSCs. Based on NTA and BCA results, around 1.0 × 10^10^ EVs equals to 100 μg EVs. The vesicle lysate and DPSC lysate were compared by western blot after adjusting the total protein concentration in each case to 10 μg/mL (Figure 2C). The transmembrane protein CD63 was significantly enriched in the vesicles, and the cytosolic proteins TSG 101 and GAPDH were also present, but the intracellular protein calnexin was not. Based on definitions provided by the International Society for Extracellular Vesicles (ISEV), the characteristics of the isolated vesicles meet the criteria for identification as EVs [4]. 

### 2.3. DPSC-Derived EVs Are Internalized by Human Umbilical Vein Endothelial Cells (HUVEC)

To determine whether the EVs are taken up by HUVECs, the EVs were labeled with the lipophilic dye CellMask Green, and then, added to cultured HUVECs for 1, 3, 6, and 12 h. The HUVECs started to internalize the EVs after 1 h, as shown by the appearance of intracellular green dots, and the number of internalized EVs increased steadily until a maximum at 6 h (Figure 2D). After 12 h, the number of green dots declined significantly, probably reflecting the loss of internalized EVs via host cell metabolism (Figure 2D). The time dependent uptake of EVs was confirmed and quantified as the integrated fluorescent density signal per cell (Figure 2E).

### 2.4. Fibrin Gels Can Be Homogeneously Loaded with EVs

**Figure 3:**
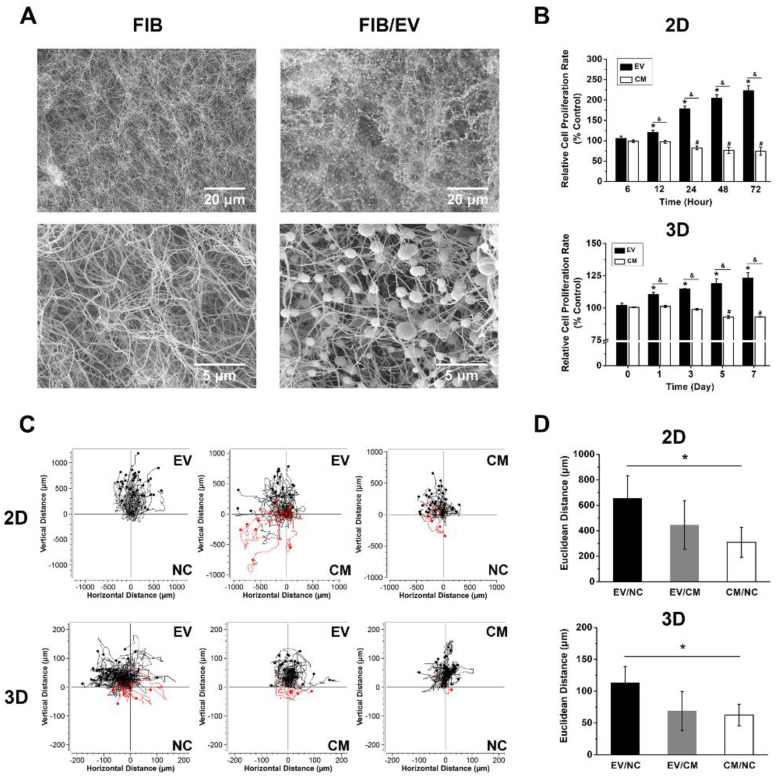
The evaluation of EV-loaded fibrin gel and its effect on cell proliferation and migration. (**A**) SEM images of fibrin gel surface and EV-loaded fibrin gel surface. (**B**) Histograms of cellular proliferation in monolayer and fibrin gel as determined by XTT assay. Please note that the 3D system comprises a co-culture of HUVEC and DPSCs with an equal number. (* *p* < 0.05 between EVs treated group and control, ^#^ *p* < 0.05 between CM treated group and control, ^&^ *p* < 0.05 between EVs and CM treated group). (**C**) Images of the plotted cellular migration path as determined by chemotaxis assay. The black lines present the HUVEC pathways towards the upper chamber (experimental group), while the red lines show the movement to the lower chamber (control). Data quantified from the experiment are shown in (**D**). The mean and standard deviation of triplicate experiments are plotted. * *p* < 0.05.

Fibrin gels were prepared with a fibrin concentration of 2 mg/mL and were loaded with EVs at a concentration of 200 μg/mL. The structures of the EV-loaded and untreated control gels were compared by scanning electron microscope (SEM), revealing that the EVs were homogeneously distributed in the gel with a high density (Figure 3A). The EVs embedded in the gel appear larger than the naked EVs, perhaps due to EV aggregation, or perhaps as an artifact of the SEM preparation steps, such as critical-point drying. 

### 2.5. DPSC-Derived EVs Enhance Cell Growth in Monolayers and within Fibrin Gels

EVs were supplied at a concentration of 25 µg/mL in the monolayers or 50 µg/mL in the gels. Conditioned medium (CM) without depletion of EVs and EBM-2 (negative control, NC) were used as two control groups. In the 2D format, the EV-treated HUVECs grew at a significantly higher rate than cells cultured in CM at 6 h (Figure 3B). Furthermore, the cells grown in CM showed significant growth inhibition after 12 h. In the 3D gels, we firstly cultured HUVECs alone in the fibrin gel, but cell proliferation declined significantly in all three groups, indicating that HUVECs cannot survive alone in fibrin gels under serum-free condition (Supplementary Figure S2). We therefore co-cultivated HUVECs with an equal number of DPSCs in the fibrin gels, with or without EVs. In the co-culture system, cell growth in the EV-loaded fibrin gels was significantly higher compared to those in bare gels since day 1, and the growth rate of the cells in the CM-treated fibrin was significantly inhibited since day 5 (Figure 3B). In 2D tests, we started the experiments with an optimal seeding density of 1.8 × 10^4^ cells per cm^2^. To avoid inhibition of cell proliferation by confluence, the experiments had to be stopped after two days. Therefore, 2D and 3D results cannot be correlated directly. 

Taken together with the internalization assay, these results show that the EVs exerted positive effects on the growth of HUVECs in monolayer culture and on 3D co-cultured HUVECs and DPSCs in EV-loaded fibrin gels. This effect lasted for seven days, suggesting that an effective dose was available over the whole observation period. 

### 2.6. DPSC-Derived EVs Enhance HUVEC Migration in Monolayers and Fibrin Gels

We tested three scenarios in monolayers and fibrin gels (Figure 3C): (1) EVs in EBM-2 versus the negative control EBM-2 without depletion of EVs (EV/NC); (2) EVs in EBM-2 versus conditioned medium (EV/CM); (3) Conditioned medium versus negative control (CM/NC). Figure 3C shows the migration pathway of HUVECs of all groups. In the 2D chemotaxis assay, HUVECs showed a strong trend towards EVs in the EV/NC group, as well as in the EV/CM group. A similar trend of migration pathway was also found in the 3D chemotaxis assay, but the migration distance was reduced. Both in 2D and 3D assays, the migration distance of EV-treated HUVECs was found to be significantly different from CM-treated HUVECs (Figure 3D).

### 2.7. DPSC-Derived EVs Induce Vascular Tube Formation in Fibrin Gels

**Figure 4:**
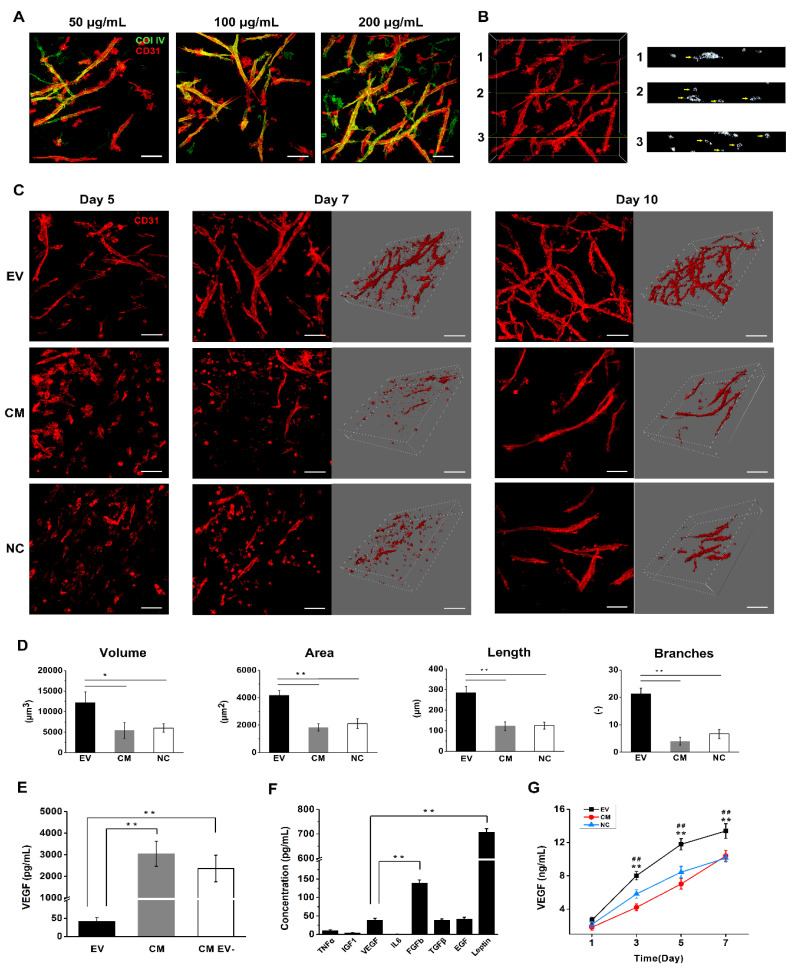
Angiogenesis assays in 3D in vitro culture. (**A**) Images of the EVs dose evaluation in fibrin gel. (Scale bar: 100 μm) (**B**) Cross-sections of image stacks showing the formation of hollow tubular-like structures in 200 µg/mL EV-loaded fibrin gel. The lumen structures were pointed by the yellow arrows. (**C**) Images of angiogenesis status in EV-loaded, CM treated, and bare fibrin gel on day 5, day 7, and day 10. (Scale bar: 100 μm) (**D**) Structure volume, surface area, structure length, and branching points were quantified as parameters. (**E**) VEGF presented in EV lysate, CM, and EV-depleted CM was detected by ELISA. Collected CM was split and EVs were isolated and quantified from one part to allow normalization of the EV concentration (approx. 10^8^ EVs/mL). The same depleted medium served as CM EV. (**F**) The angiogenic factors presented in EV lysate were further explored by ELISA. (**G**) The release of VEGF from co-cultures of DPSCs and HUVECs in hydrogels was measured over seven days by ELISA. Cells were cultured in EV-loaded fibrin gel (EV, basal EGF-2) and bare fibrin gels covered with basal EGF-2 (NC) and conditioned medium (CM), (* statistically significant results compared with the CM group, # compared with the NC group). The mean and standard deviation of triplicate experiments are plotted. * *p* < 0.05, **/## *p* < 0.01.

EVs were previously shown to promote vascularization in a dose-dependent manner in monocultures [5,17]. To determine whether this result could be replicated in our system, we optimized the concentration of EVs in the fibrin gels. After seven days under serum-starved culture, co-cultured DPSCs and HUVECs formed tubular structures in a dose-dependent manner in fibrin gels containing increasing concentrations of EVs, with the most extensive tubular network forming at an EV concentration of 200 μg/mL (Figure 4A). In this optimized setup, most HUVECs were aligned, reoriented and sprouted to form new vessels, whereas only a few cells remained in clusters. Surrounding the tubular structures, large amounts of collagen IV were deposited, which is a key component of the vascular basement membrane and may indicate the maturation of vascular structures [18]. Cross-sections of two-photon laser-scanning microscopy (TPLSM) image stacks revealed the formation of a lumen with a diameter of 5–30 μm, thus, confirming vascular maturation (Figure 4B). HUVECs in the EV-loaded fibrin gel began to align and reorient on day 5, and showed extensive branching on day 10 (Figure 4C). In contrast, most HUVECs in the CM and NC samples remained rounded and clustered on day 7, and fewer tubules were present on day 10. The volume, surface area, length, and number of branch points in the tubular structures were evaluated to provide a quantitative analysis of each system, revealing significantly higher values for all four parameters in the EV-loaded fibrin gel compared to the CM and NC groups (Figure 4D). This confirms that the EV-loaded fibrin gel has a much greater proangiogenic potential than the bare fibrin gels. 

### 2.8. DPSC-Derived EVs Contain VEGF and Several Additional Proangiogenic Factors 

We measured the concentration of VEGF in EV lysate, CM and EV-depleted CM by ELISA, because this growth factor is the key mediator of angiogenesis. Interestingly, the EVs contained a significantly lower amount of VEGF than the other samples (Figure 4E). We therefore measured the level of other proangiogenic factors in the EV lysate, revealing that FGFb and leptin were present at significantly higher levels than VEGF; TGFβ and EGF were present at similar levels to VEGF; TNFα and IGF1 were present at low levels; IL6 was not detected (Figure 4F). The quantification of mentioned proangiogenic factors was shown in Supplementary Tables S1 and S2. 

### 2.9. EV-Loaded Fibrin Gels Promote the Secretion of VEGF

The release of VEGF from the hydrogels, containing a co-culture of HUVECs and DPSCs was measured by ELISA over a period of seven days (Figure 4G). In the EV-loaded fibrin gel, there was an initial steep increase in VEGF release, which lasted for three days, followed by a slightly lower release rate until day 5 and a subsequent transition to a plateau phase until day 7. From day 3, the detected VEGF release from co-cultures in EV-loaded fibrin gels was significantly higher than from co-cultures in fibrin gels without EVs (NC, both groups covered with EBM-2) and from co-cultures in fibrin gels without EVs but covered with conditioned medium (CM). The release of VEGF from the co-culture in EV-loaded gels and the NC sample was characterized by a rapid increase and slow flattening, while the release of VEGF from the CM sample was characterized by a slow and gentle increase over the whole monitoring period.

### 2.10. EV-Loaded Fibrin Gels Stimulate the Deposition of Collagen I and III

**Figure 5:**
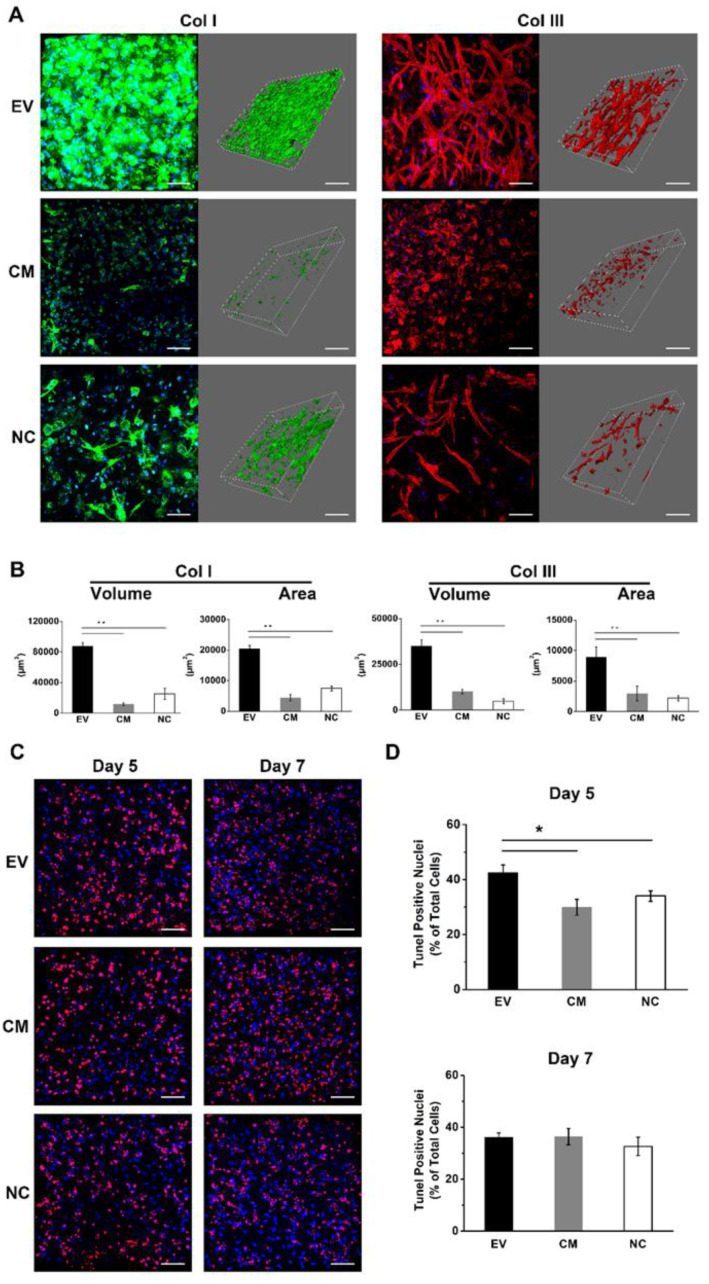
Extracellular matrix and apoptosis assays in 3D in vitro culture. (**A**) Images of collagen deposition in EV-loaded, CM-treated and bare fibrin gels on day 7. (Scale bar: 100 μm) Structure volume and surface area were quantified as parameters and are shown in (**B**). (**C**) Apoptosis was examined in situ using a TUNEL assay. (Scale bar: 100 μm) Quantitative data are shown in (**D**). The mean and standard deviation of triplicate experiments are plotted (* *p* < 0.05, ** *p* < 0.01).

The deposition of ECM was investigated by immunohistochemical staining of collagen I and III. We found that both proteins were deposited after the cultivation of HUVECs together with DPSC in EV-loaded fibrin gels for seven days (Figure 5A). Collagen I was homogenously distributed in the gel, whereas collagen III was deposited along with the tubular structures. In contrast, the CM and NC group showed little evidence of collagen I deposition, and collagen III was distributed as amorphous deposits. The EV-loaded fibrin gel also showed significantly higher values for collagen I and III structure volume and surface area compared to the CM and NC groups (Figure 5B). 

### 2.11. EV-Loaded Fibrin Gels Accelerate the Rate of Apoptosis during Initial Angiogenesis

Vascularization and angiogenesis involve extensive apoptosis [19], so we evaluated the frequency of apoptosis in the fibrin gel using a terminal deoxynucleotidyl transferase dUTP nick end labeling (TUNEL) assay. Apoptotic cells were stained red with bromodeoxyuridine and the nuclei were counterstained with DAPI (Figure 5C). On day 5, the EV-loaded fibrin gel showed a significantly higher proportion of apoptotic cells relative to the total cell count compared to the CM and NC groups (Figure 5D). However, there were no significant differences among the three groups on day 7. 

## 3. Discussion

In vitro culture of endothelial cells usually requires serum and various growth factors. In this study, we used basal media without any exogenous growth factors, in order to simulate the condition of nutritional deficiency during pulp regeneration. When DPSCs and HUVECs were co-cultured, the EV-loaded fibrin gel promotes rapid vascularization, including increased collagen matrix deposition, accelerated apoptosis and the release of VEGF. The EV-loaded fibrin gels therefore provided excellent functional and structural support for vascularization. When embedded within such gels, the biological activity of the EVs lasted over the whole observation period of seven days, confirming that the fibrin gel retained EVs and preserved their activity. 

The role of MSCs-derived EVs in the modulation of vascular development, growth, and maturation has been well studied in bone marrow-derived (BM-MSC) [20], adipose-derived (AD-MSC) [21], and human umbilical cord mesenchymal stromal cells (HUMSC) [22]. The beneficial effects of DPSC-derived EVs are seldom explored and only recent studies have demonstrated them. Xian et al. [7] investigated the mechanisms involved in angiogenesis by DPSC-derived EVs and showed that EVs inhibited the p38 MAPK signaling pathway to enhance tubular morphogenesis. The results of this investigation support the findings of our study. Merckx et al. [23] compared the proangiogenic effects of BM-MSC and DPSC-derived EVs and emphasized the importance of the choice of cell type. While in this study, DPSC- and BM-MSC-derived EVs contained a plethora of angiogenesis-related proteins and exerted a chemotactic effect on endothelial cells, they only partially contributed to inducing angiogenesis. Especially for the DPSC-derived EVs, the authors could not find a significant effect in vitro and in vivo. Nonetheless, it is well known that just minor differences in the used methods, e.g., for isolation and cultivation of the cells, can have a significant influence on the functional properties of the isolated EV fraction. 

Previous studies mostly underlined the biological effects of EVs in monolayer cultures, however, our study aims at exploring the effects in 3D cell environments, since cells in 3D cultures behave differently compared to monolayer cultures [24]. Herein, the combination EVs within fibrin gel offer several advantages for the regeneration of dental pulp. For example, the system is convenient for clinical use because it can be administered using a dual-barrel syringe to induce spontaneous cross-linking reactions in situ [25]. Given a gelation time of less than 30 s, the injectable fibrin gel can easily be used to fill irregular-shaped defects with a minimally invasive surgical procedure. The fiber density, fibrin diameter, and pore size of the gel can easily be adjusted by varying the concentration of fibrinogen, providing precise control of the gel microstructure for the improvement of cell growth, nutrition and the supply of growth factors [26]. 

Although it has been shown that EVs only achieve the intended biological effects by internalization through target cells based on an endocytic pathway [27], in principle, cell entry is not necessary to achieve a biological effect. Thus, the effect may be induced by surface binding and downstream signaling, or the vesicles may collapse and release their bioactive cargo into the cell environment [28]. For tumor-derived exosomes (TEX), which play an important role in the promotion of tumor angiogenesis, it has just recently been shown that a growth stimulation of endothelial cells (ECs) in vitro and an angiogenic effect in vivo was mainly mediated by the adenosine A_2B_ receptor. In this study, TEX were clearly identified as carrier and producer of adenosine [29]. In principle, this could also apply to DPSC-derived EVs. Future studies will be necessary to fully understand the cargo of the DPSC-derived EVs, their interaction with the recipient cells and mode of action. The results of the present work can only give an impetus for this.

In our EV-loaded fibrin gel, it was found that the fibrin fibers surround the EVs and bind firmly to the surface. We believe that such a structure allows effective interaction between cells and EVs in a 3D environment. It is also noted that EVs may have the ability to recruit adjacent cells. An indication of this is provided by the results of the migration assays in this study.

Co-cultures of endothelial cells with supporting cells are often used to generate microvasculature and form stable networks in engineered tissue grafts [30]. Previous work by our group and others has shown that it typically takes 14 days for co-encapsulated endothelial cells and supporting cells to assemble into 3D vascular-like structures in vitro [31,32,33]. Given that direct anastomosis between the engineered microvascular network and host vasculature does not occur in dental root canals, it is impossible for implanted 3D constructs to survive long enough for vascularization, and the process must therefore be accelerated. This can be achieved by incorporating growth factors into the supporting scaffold, but many growth factors are soluble and unstable proteins, reducing the efficiency of this approach [34]. Furthermore, the uncontrolled release of proangiogenic factors promotes tumorigenesis [35]. We therefore used EVs instead of soluble growth factors, allowing the provision of multiple growth factors simultaneously, and also ensuring that these vulnerable bioactive contents were protected by the EV rigid bilayer membrane. The direct uptake of EVs by target cells ensures that the encapsulated growth factors are never exposed to the extracellular environment, and the rate of uptake by endocytosis could be controlled by varying the composition of the EV-loaded fibrin gel.

Among the multiple proangiogenic factors detected in the EVs, we found that VEGF, the key mediator of angiogenesis, was much less abundant in the EV lysate than in CM. This finding matches with the results of Merckx and co-workers [31]. This study also showed just low VEGF values in lysed DPSC-EVs compared to EV-depleted CM. In addition, the protein expression of non-lysed EVs was also tested and described as negligible, which proves that the tested factors were intravesicularly localized and not membrane-associated or precipitated during ultracentrifugation. This particular investigation was not performed in our study.

VEGF is the key mediator of angiogenesis and contributes to stimulate blood vessel formation [36]. Even so, the EV-loaded fibrin gel induced the formation of a significantly higher density of vascular-like structures than gels treated with CM when co-culturing DPSCs and HUVECs, in part by triggering the sustained release of VEGF over several days. Since the EVs do not include abundant VEGF, it is possible that EVs boost the encapsulated cells to secrete VEGF so that it supports rapid vascularization. TNFα, IGF-1, TGFβ and EGF, which are MSCs paracrine factors and involved in vascular regeneration, were also detectable but at low concentrations (<50 pg/mL). Comparatively high levels of leptin and FGFb were determined in EV lysate, which had previously been shown to act synergistically with VEGF to induce angiogenesis [37]. This is of special interest, because there is direct evidence that leptin and VEGF expressed in ameloblasts and odontoblasts during tooth development induce angiogenesis in tooth germs during development [38]. Furthermore, leptin has been shown to induce the upregulation of VEGF, odontogenic differentiation and mineralization in human dental pulp cells in vitro [39]. It can be speculated that leptin is perhaps a tooth specific promoter of angiogenesis. 

Since the proangiogenic factors usually promote cell differentiation above the concentration of 1 ng [40,41], we could conclude that EVs contain mainly transmembrane proteins, but soluble proteins, such as VEGF and FGFb, are not regularly encased into EVs. miRNAs in EVs possibly contribute a more important role to stimulate VEGF secretion and vascular regeneration [42,43]. 

Angiogenesis requires the deposition of ECM components to support the mature neovasculature. The most abundant protein components of the ECM are collagens, and angiogenesis therefore tends to correlate with the accumulation of collagen. Accordingly, we found that collagen IV (a major component of the basement membrane) had accumulated along the new vessels in the EV-loaded fibrin gel by day 7, and collagen I and III (which confer mechanical stability) were also expressed at high levels. Collagen is the main component of the connective tissue ECM [44], and the combination of collagen I and III were found in healthy pulp tissue [45], indicating the early-stage pulp-like tissue formation in parallel with rapid vascularization in our findings. We also observed a higher frequency of apoptosis among HUVECs in the EV-loaded fibrin gel by day 5, compared to the CM and NC gels, but there were no significant differences among the three groups by day 7. These results suggest that the EVs induced apoptosis to control the maturation of vascular structures in the 3D gels but that the process was mostly complete after seven days. Apoptosis helps to reduce the number of endothelial cells during vessel network reorganization; otherwise, the uncontrolled angiogenesis stimulates tumorigenesis [46]. 

Although the EV-loaded fibrin gel was shown to promote angiogenesis, the low yield of DPSC-derived EVs is a major challenge because the current method would not be suitable for larger tissue defects. It would also be difficult to standardize a clinical approach without refinement to ensure batch-to-batch consistency between different preparations of EVs. Even so, we conclude that we have successfully isolated and characterized DPSC-derived EVs and prepared EV-loaded fibrin gels using a simple dual-barrel syringe to promote spontaneous cross-linking reactions in situ. Such gels combine the structural and biochemical properties required to induce the rapid vascularization of engineered dental pulp, including the deposition of ECM components and the promotion of apoptosis. These findings not only offer insight into the proangiogenic effects of DPSC-derived EVs in 3D culture, but also demonstrate a practical and non-invasive method for the rapid regeneration of dental pulp. The detailed molecular basis of rapid vascularization induced by DPSC-derived EVs will be considered in our future studies. 

## 4. Materials and Methods 

### 4.1. Cell Culture and Characterization

Healthy human third molars, extracted during orthodontic or prophylactic procedures, were collected by the Department of Oral and Maxillofacial Surgery (RWTH Aachen University Hospital, Aachen, Germany), as approved by the local ethics committee (EK 084/17, 30th March 2017). The teeth were stored in cold sterile buffer (140 mM NaCl, 4 mM KCl, 11 mM D-glucose, 10 mM Hepes, pH 7.4) for no more than 24 h before the start of isolation procedure. Teeth were cleaned and split by a hammer. The pulp tissue was obtained from the crown and roots and minced into 1–2 mm segments. Pulp tissue from different teeth was subjected separately and digested in 1 mL enzyme solution (3 mg/mL collagenase I and 4 mg/mL dispase II, both from Gibco, Carlsbad, CA, USA) for 1 h at 37 °C. The isolated cells were seeded in a 25 cm^2^ flask and cultivated in Alpha Modified Eagle’s medium (α-MEM) containing 10% fetal bovine serum (FBS) and 1% antibiotics/antimycotics (all reagents from Gibco, Carlsbad, CA, USA). The cells were split by trypsinization when they reached approximately 80% confluency. Cells up to passage 6 were used for our experiments. DPSCs were identified by flow cytometry (BD Biosciences, San Jose, CA, USA) based on the detection of the surface markers CD90, CD73, CD105, CD79a, CD45, CD11b, CD34 and HLA-DR.

Endothelial cells were isolated from human umbilical cord veins (HUVECs) provided by the Department of Gynecology and Perinatal Medicine (RWTH Aachen University Hospital, Aachen, Germany) as approved by the local ethics committee (EK 084/17, 30th March 2017). The umbilical cord vein was prepared and flushed with phosphate-buffered saline (PBS, Gibco, Carlsbad, CA, USA) before removing the HUVECs using 1 mg/mL collagenase II (Gibco, Carlsbad, CA, USA). The isolated cells were seeded in flasks precoated with 2% type B gelatin (Sigma-Aldrich, Darmstadt, Germany) and were cultivated in endothelial cell growth medium (EGM-2, PromoCell, Heidelberg, Germany). Cells up to passage 4 were used for our experiments. HUVECs were verified by immunohistochemical staining for CD31 (also shown in Figure 2 and Figure 4) and vWF.

### 4.2. Isolation of DPSC-Derived Extracellular Vesicles

Exosome-depleted α-MEM was prepared by ultracentrifugation at 110,000× *g* for 5 h. DPSCs (passages 3–6) were cultured in exosome-depleted α-MEM for 72 h after the last medium exchange. Then, the supernatant was collected as the conditioned medium (CM) and used as control in the subsequent experiments. EVs were harvested from CM by differential centrifugation. Briefly, dead cells, cell debris, and large vesicles were removed by centrifugation at 300× *g* for 10 min, 2000× *g* for 20 min, and 10,000× *g* for 40 min. The supernatant was collected and the EVs were pelleted by ultracentrifugation using an Optima LE-80K ultracentrifuge with an SW 32 Ti rotor (Beckman Coulter, Chaska, MN, USA) at 110,000× *g* for 90 min. The pellet was washed with PBS and a second round of ultracentrifugation was carried out as above. The total protein content of the EVs was determined using a BCA Protein Assay Kit (Thermo Fisher Scientific, Waltham, MA, USA).

### 4.3. Identification of DPSC-Derived EVs

The presence of EVs was confirmed by TEM. The pelleted EVs were suspended in double-distilled water and applied dropwise onto formvar carbon-coated nickel grids. The grids were air-dried, stained with 2% uranyl acetate and viewed under a Zeiss LEO 906E transmission electron microscope (Zeiss, Jena, Germany). 

The size range and concentration of EVs were determined by NTA using a NanoSight NS300 (Malvern Instruments, Worcestershire, UK). EV pellets were diluted to ensure that the concentration of detectable particles was within the optimal range of 10^7^–10^9^ particles/mL. For data acquisition, each sample was recorded using auto-detectable settings and five 60 s videos with NTA software (Malvern Instruments, Worcestershire, UK). 

EV markers were detected by western blot. DPSCs and EV pellets were lysed in RIPA buffer containing a protease inhibitor cocktail (Roche, Basel, Switzerland). The lysates were separated by SDS-PAGE (10% acrylamide) and transferred onto nitrocellulose membranes. After blocking with 5% skim milk, the blots were incubated overnight at 4 °C with the primary antibodies anti-CD63 (Invitrogen, Carlsbad, CA, USA), anti-TSG-101 (Sigma-Aldrich, Darmstadt, Germany), anti-calnexin (BD Biosciences, San Jose, CA, USA) and anti-GAPDH (Invitrogen, Carlsbad, CA, USA), followed by incubation with secondary antibodies conjugated to horseradish peroxidase (Cell Signaling Technology, Danvers, MA, USA) at room temperature for 1 h. The signals were developed by electrochemiluminescence (ECL) detection (Thermo Fisher Scientific, Waltham, MA, USA) and quantified using a chemiluminescence detector (Analytik Jena, Thuringia, Germany).

### 4.4. EV Labeling and Internalization Assay

DPSC-derived EVs were labeled with CellMask Green dye (Invitrogen, Carlsbad, CA, USA) for 30 min at room temperature in 200 μL PBS in the dark. Excess dye was removed by ultracentrifugation at 110,000× *g* for 90 min in 38 mL PBS. We then added 50 µg of the labeled EVs to HUVECs, which were cultivated at a density of 0.5 × 10^6^ cells per well in six-well plates. The medium was removed at specific time points and the cells were fixed in 4% paraformaldehyde prior to staining with CD31 diluted 1:100, Alexa Fluor 594 diluted 1:400, and DAPI (all antibodies from Thermo Fisher Scientific, Waltham, MA, USA). The samples were observed using an AxioObserver Z1 fluorescence microscope (Zeiss, Jena, Germany). The number of internalized EVs was quantified as integrated fluorescent density signal per cell by ImageJ software (Adobe Systems Inc., San Jose, CA, USA).

### 4.5. Preparation and Characterization of EV-Loaded Hydrogels 

For hydrogel preparation, fibrinogen from human plasma (Merck Millipore, Burlington, MA, USA) was diluted in Tris-buffered saline (TBS) to an initial stock concentration of 4 mg/mL. In total, 10, 20, or 40 μg EVs were suspended in 100 µl of the fibrinogen stock solution. Cell suspensions were added to a polymerization solution containing 30 U/I thrombin from bovine plasma (Sigma-Aldrich, Darmstadt, Germany) and 4.75 mM calcium chloride. The cell-loaded polymerization solution was then mixed 1:1 with the EV-loaded fibrinogen stock solution. The final concentration of fibrin gel contains 2 mg/mL fibrinogen with 50, 100, or 200 μg/mL EVs. The EV-loaded fibrin gels were fixed in 3% glutaraldehyde, washed in PBS, and dehydrated by incubation in solutions containing progressively higher proportions of acetone. After critical-point drying with CO_2_, the samples were sputter-coated with a 20 nm gold-palladium layer and images were acquired by SEM using an ESEM XL 30 FEG microscope (FEI/Philips, Hillsboro, OR, USA).

### 4.6. Cell Proliferation Assay

The proliferation of HUVECs with and without EVs was tested in monolayer cultures and in 3D fibrin gels using an XTT assay kit (Roche, Basel, Switzerland). Both in the 2D and 3D tests, we performed three groups: EBM-2 with EVs, CM and EBM-2 (control group). In the 2D format, the HUVECs were seeded in 96-well plates at a density of 6000 cells/well and cultivated in serum-free medium overnight. Then, 100 µL CM and EBM-2 with or without EVs (25 µg/mL) were added to the wells. The proliferation of HUVECs was monitored after 3, 6, 12, 24 and 48 h. In the 3D format, we initially cultured HUVECs alone with or without EVs (25 µg/mL) in fibrin gel, but the results showed remarkably decreased cell proliferation during the culture period in all three groups (Supplementary Figure S2). Hence, we co-cultured HUVECs 1:1 with DPSCs in 96-well plates at a density of 12,000 cells in 40 μL fibrin gels/well with or without EVs (50 µg/mL). Proliferation was monitored on days 1, 3, 5 and 7. 

### 4.7. Chemotaxis Assay

The migration of EV-treated HUVECs in the 2D and 3D systems was evaluated using a chemotaxis assay kit according to the manufacturer’s protocol (Ibidi, Gräfelfing, Germany). Briefly, 6 μL of HUVEC suspension (5 × 10^6^ cells/mL) was seeded into the center chamber of a µ-slide. A thin channel connected the center chamber to two opposing medium reservoirs. The experiment was set up with three groups each for the 2D and 3D systems: EVs in EBM-2/negative control (EBM-2), CM without depletion of EVs/negative control and EVs in EBM-2/CM without depletion of EVs (Supplementary Figure S3). The concentration of EVs used in chemotaxis assay was 25 µg/mL. Cells were visualized on a JuLI Stage real-time cell history recorder (NanoEnTek, Seoul, Korea). Cell movement was monitored every 10 min for 48 h, and cell tracks were plotted in ImageJ. 

### 4.8. Angiogenesis Assay

Three different HUVEC/DPSC co-culture systems were established: (1) EV-loaded gels containing co-cultured cells cultivated in EBM-2; (2) gels containing co-cultured cells but no EVs cultivated in EBM-2; (3) gels containing co-cultured cells but no EVs cultivated in an 1:1 mixture of EBM-2 and CM. The two cell types were prepared individually at concentrations of 6 × 10^6^ cells/mL, mixed at a 1:1 ratio and applied to the gels at a concentration of 1.2 × 10^6^ mixed cells per 200 μL of fibrin gel. The medium (EBM-2 or CM/EBM-2) was supplemented with 0.16 mg/mL tranexamic acid (Pfizer, New York, NY, USA). Samples were analyzed by TPLSM on days 5, 7 and 10 to visualize vascular and collagen structures using an Olympus FluoView FV1000 MPE microscope (Olympus, Hamburg, Germany). After being fixed in 4% paraformaldehyde for 2 h, the hydrogel samples were stained with primary and secondary antibodies. Information about the antibodies is listed in Supplementary Table S3. Each sample was imaged at random locations with a stack depth of 80 μm using a 25× magnification water immersion lens (Olympus XLPLN 25 × WMP-SP, NA 1.05, Hamburg, Germany). The dimensions of the 3D stacks were set to 500 × 500 × 80 μm. Imaris imaging software (Bitplane, Zurich, Switzerland) was used to analyze the 3D stacks. 

### 4.9. Apoptosis Assay

The proportion of apoptotic cells in each sample was determined using a TUNEL assay kit (Abcam, Cambridge, UK) and counterstained with DAPI. TUNEL-positive cells were detected by TPLSM as described above. 

### 4.10. Release of Angiogenic Factors

The proangiogenic factors from the EV lysate and CM were quantified by ELISA (Signosis, Santa Clara, CA, USA). At scheduled time intervals, the supernatants of co-cultured HUVECs and DPSCs were collected, replaced with an equal volume of fresh medium, and analyzed for secreted VEGF by ELISA kit (R&D Systems, Minneapolis, MN, USA).

### 4.11. Statistical Analysis

Statistical analysis was carried out using GraphPad Prism v5 (GraphPad Software, San Diego, CA, USA). Quantitative data are presented as means ± standard deviations. Between-group differences were evaluated using Turkey’s *t*-test or one-way analysis of variance (ANOVA), with *p* values below 0.05 considered statistically significant.

## Supplementary Materials

Supplementary materials can be found at https://www.mdpi.com/1422-0067/21/12/4226/s1.

## Abbreviations

AD-MSCs	Adipose-derived mesenchymal stromal cells
BM-MSCs	Bone marrow-derived mesenchymal stromal cells
CO_2_	Carbon dioxide
CM	Conditioned medium
DPSCs	Dental pulp stem cells
ECM	Extracellular matrix
ELISA	Enzyme-linked immunosorbent assay
EVs	Extracellular vesicles
HUMSCs	Human umbilical cord mesenchymal stromal cells
HUVECs	Human umbilical cord veins
miRNA	microRNA
mRNA	messenger RNA
MSCs	Mesenchymal stromal cells
NTA	Nanoparticle tracking analysis
SEM	Scanning electron microscopy
TEM	Transmission electron microscopy
TPLSM	Two-photon laser-scanning microscopy
VEGF	Vascular endothelial growth factor
