# Supplementary material for: Extracellular Vesicles-Loaded Fibrin Gel Supports Rapid Neovascularization for Dental Pulp Regeneration
Source: Int J Mol Sci. 2020 Jun 13;21(12):4226. doi: 10.3390/ijms21124226 (PMC7352754; doi:10.3390/ijms21124226)
Supplement: Supplementary file 1 [file ijms-21-04226-s001.pdf]

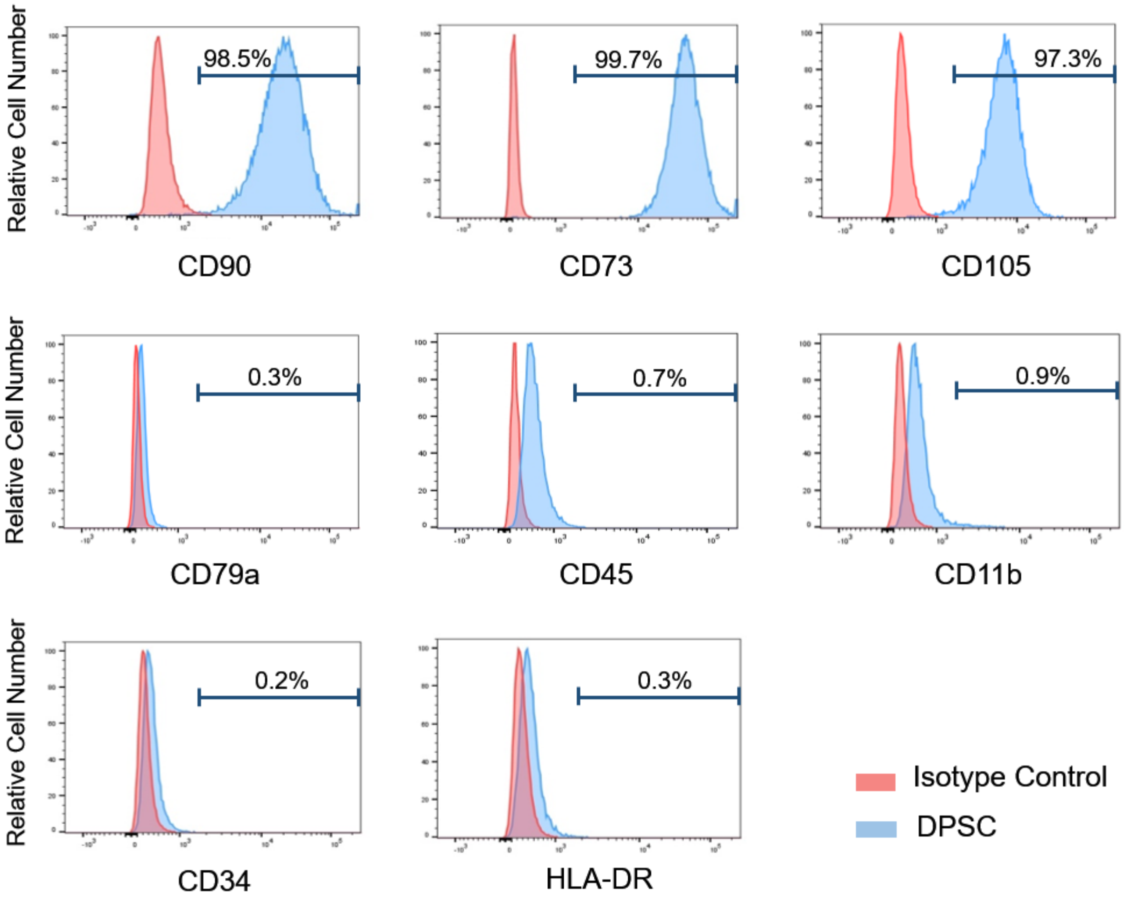


**Supplementary Figure S1.** Flow cytometry analysis of the immunotype of DPSCs. The mesenchymal markers (CD73, CD105) and the cell adhesion molecules (CD90) were positive, whereas the hematopoietic lineage markers (CD45, CD34, CD11b, CD79a and HLA-DR) were negative. Please note: Multicolor approach caused a slight shift in the histograms for CD45 and CD11b due to a spill over. Stated values represent the positive fraction after compensation calculation.

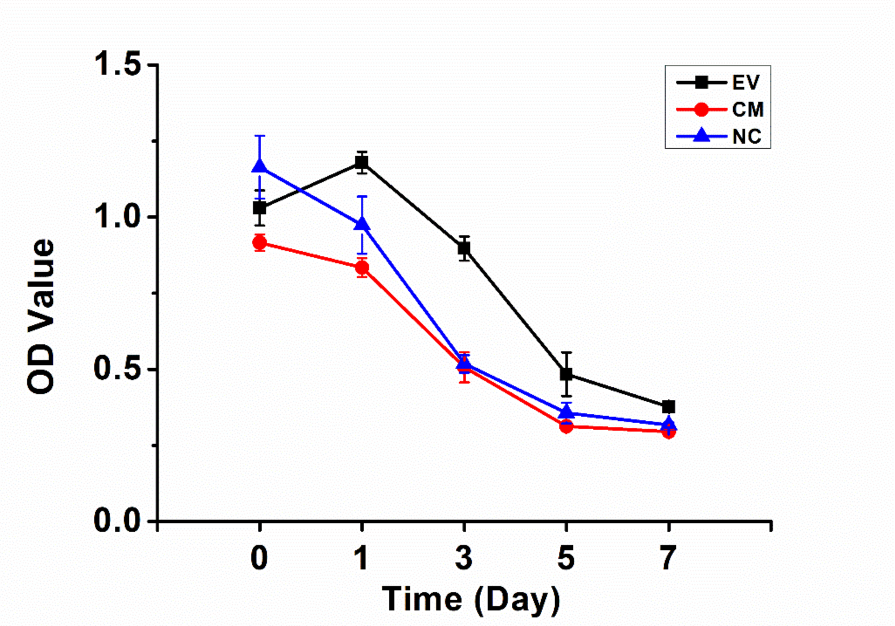


**Supplementary Figure S2.** The XTT test of HUVECs grew in fibrin gels alone.

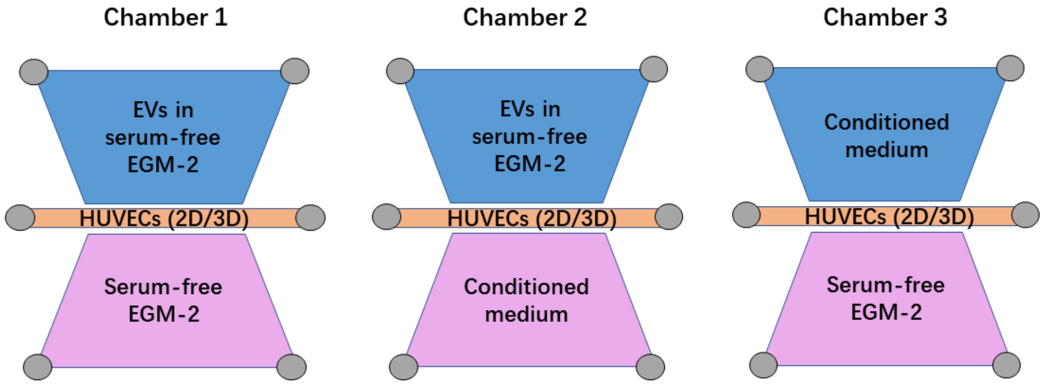


**Supplementary Figure S3.** The schematic diagram of chemotaxis assay set up. The Ibidi µ-slide has a thin channel connecting the center chamber to two opposing medium reservoirs. The experiment was set up with three groups each for the 2D and 3D systems: EV/negative, CM/negative and EV/CM in EGM-2.

**Supplementary Table S1.** VEGF concentration in EV lysate, CM and EV-depleted CM (pg/mL, mean ± S.E.M.).

| | VEGF Concentration |
|---|---|
| EV lysate | 36.1 ± 2.8 |
| CM | 5447.4 ± 82.3 |
| EV-depleted CM | 4768.7 ± 115.9 |

**Supplementary Table S2.** Protein concentration in EV lysate (pg/mL, mean ± S.E.M.).

| | TNFα | IGF1 | VEGF | IL6 | FGFb | TGFβ | EGF | Leptin |
|---|---|---|---|---|---|---|---|---|
| EV lysate | 10.8 ± 1.0 | 4.46 ± 0.3 | 34.6 ± 2.2 | not detectable | 138.8 ± 4.6 | 38.9 ± 1.0 | 41.1 ± 2.3 | 707.4 ± 6.5 |

**Supplementary Table S3.** Antibodies List.

| Antibody | Company | Dilution | Clone Number | Catalog Number |
|---|---|---|---|---|
| Primary Antibody | | | | |
| mouse anti-CD31 | Sigma | 1:100 | Mouse monoclonal, WM-59 | P 8590 |
| rabbit anti-collagen I | Acris | 1:100 | Rabbit polyclonal | R 1038 |
| rabbit anti-collagen III | Acris | 1:100 | Rabbit polyclonal | R 1040 |
| rabbit anti-collagen IV | Acris | 1:100 | Rabbit polyclonal | BP 5031 |
| Secondary Antibody | | | | |
| goat anti-mouse Alexa Fluor 594 | Thermo Fisher Scientific | 1:400 | Goat polyclonal | A 11005 |
| goat anti-rabbit Alexa Fluor 594 | Thermo Fisher Scientific | 1:400 | Goat polyclonal | A 11012 |
| goat anti-rabbit Alexa Fluor 488 | Thermo Fisher Scientific | 1:400 | Goat polyclonal | A 11008 |
